# A case report involving the experience of pervasive pregnancy denial: detailed observation of the first 12 postpartum weeks

**DOI:** 10.1186/s12888-022-04377-1

**Published:** 2022-12-09

**Authors:** Natalia Chechko, Elena Losse, Susanne Stickel

**Affiliations:** 1 Department of Psychiatry, Psychotherapy and Psychosomatics, Faculty of Medicine, RWTH Aachen, Pauwelsstraße 30, 52074 Aachen, Germany; 2 Institute of Neuroscience and Medicine: JARA-Institute Brain Structure Function Relationship (INM 10), Research Center Jülich, Jülich, Germany; 3 Institute of Neuroscience and Medicine, Brain & Behavior (INM-7), Research Center Jülich, Wilhelm-Johnen-Strasse, 52428 Jülich, Germany

**Keywords:** Case report, Pregnancy denial, Postpartum wellbeing, Longitudinal follow-up

## Abstract

**Background:**

Pervasive pregnancy denial is a rare condition associated with distress and unassisted delivery.

**Case presentation:**

The case involves a 38-year-old woman (NN), with two older children (ages 8 and 11), who was unaware, until delivery, that she had been pregnant. The case is discussed in the context of a 12-week observation of postpartum mood, stress, and mother-child attachment. NN and other 558 non-depressed women (mean age 32.41 years) were selected from the pool of participants in the RIPOD (risk of postpartum depression) study. All participants were recruited within 1–6 days of delivery. In addition to surveying depressed mood at childbirth, remote assessments of mood, mother-child attachment, and perceived stress were conducted at 3, 6, 9, and 12 weeks postpartum. Every other day, the participants also reported their current perceived stress levels based on a scale from 1 (low) to 10 (high). During the entire period of postpartum observation, NN reported no symptoms on the Edinburgh Postpartum Depression Scale, similar to only 1.6% of the sample, no stress as 0.7% of the sample, and above-average mother-infant bonding akin to only 4.6% of the sample. Her daily stress levels showed no disturbance, which was the case for only 3.32% of the total sample. On the day of delivery, NN reported a stress level of 1 (the minimum possible level), which was reported by only 4.2% of the total sample. However, NN reported the experience of delivery to be traumatic given that the child had fallen to the floor.

**Conclusion:**

The experience of a denied pregnancy did not appear to disturb NN at any time point, not even on the day of delivery. Compared to NN, the other non-depressed participants reported wide fluctuations in stress levels during the observation period. NN did not report any risk factors for denied pregnancy. Thus, she belonged neither to any group of typical pregnancy deniers, as reported in the literature, nor to a typical postpartum group. We postulate, therefore, that the extent to which pregnancy denial can be deemed a normal variation, unrelated to a psychological or physiological condition, depends largely on personal traits.

**Supplementary Information:**

The online version contains supplementary material available at 10.1186/s12888-022-04377-1.

## Background

Pregnancy denial, also known as cryptic pregnancy, is a phenomenon in which a mother-to-be is unaware of being pregnant. Unlike a concealed pregnancy, of which the woman is aware but does not willingly disclose it to anyone, the term cryptic pregnancy refers to a woman’s subjective lack of knowledge of being pregnant. The condition can last for several months, and, in some cases, for the entire period of pregnancy. Occurring in 1:475 births, the phenomenon is far more common than previously thought. In about 1:2455 births, a viable foetus is born without the mother being aware of her pregnancy until childbirth [1].

Miller [2] has described three types of pregnancy denial: psychotic, affective, and pervasive. In psychotic denial, physiological changes are identified falsely either as a blood clot or cancer, or foetal movements are misinterpreted as loose organs. In affective denial, on the other hand, the acknowledgment of pregnancy lacks an emotional component, enabling the pregnant woman to continue her life as though she were not pregnant [2]. It is, however, the third type of pregnancy denial that has attracted the greatest interest. Women with a pervasive denial of pregnancy appear to be completely unaware of the existing pregnancy. In this case report, we discuss this particular form of pregnancy denial in the context of the available data, suggesting that the condition cannot be attributed to any one reason.

Women with pregnancy denial have been found to belong to a heterogeneous constituency, precluding any clear explanation as to why they deny their pregnancy or what type of women may be prone to pregnancy denial [3]. According to a recent study, being young, single, socio-economically vulnerable, or using pill-based contraception can lead to pregnancy denial in some women [4]. Interestingly, psychiatric symptoms, which have been found to be linked to only a small number of these cases, are not an important feature of pregnancy denial, there being no specific psychiatric correlate of cryptic pregnancy. For instance, in an Austrian sample of 27 cases [5], 48% met the criteria for psychiatric disorders following a structured interview, although the diagnoses were highly variable: schizophrenia (7%), depression (15%), personality disorders (15%) and mild mental retardation (11%). A significantly different picture emerges from the German prospective study [6], which, having identified 62 cases of pregnancy denial, diagnosed only 5% of them as having schizophrenia, and found signs of personality disturbances or mental retardation in another 8% following an unstructured clinical interview.

While mothers seem to be generally capable of accepting and taking care of the newborn after a cryptic pregnancy, with many feeling remorse for having put their infants at risk prior to diagnosis [6, 7], some studies have indicated elevated risks with respect to neonatal outcome, in some cases resulting in neonaticide, following pregnancy denial [8]. However, cryptic pregnancies leading to neonaticide are exceptional cases of pregnancy denial, constituting a special subtype that cannot be directly compared to the cryptic pregnancies that are not associated with harmful maternal behavior toward the child. Pregnancy denials that lead to neonaticide appear to have a history of childhood trauma as a predisposing factor [9–12]. Dissociation has been found to be a consistent symptom in pervasive pregnancy denial linked to harmful maternal behavior toward the child [13].

As regards the reasons behind pregnancy denial, especially the type that leads to neonaticide, Amon et al. [12] have suggested that behaviors linked to this condition are more related to dissociative disorders or complex post-traumatic stress disorder than to personality disorders. Being pregnant puts traumatic memories at risk of returning, causing the mind to repress the somatic cues of the pregnancy [14]. In this context, the denial of pregnancy can be seen as an emotion-driven strategy in a situation that is perceived to be unalterable [2] and has to be coped with through avoidance [15]. For those whose cultural or familial beliefs prohibit sexuality, or who may have suffered sexual trauma, the reality of being pregnant can be insufferably difficult [2].

Other studies have sought to explain pregnancy denial as a consequence of the parent-offspring conflict. According to this evolutionary conflict theory, different viewpoints exist on the optimal parental investment between parents and offspring, with the latter demanding more than what is willingly made available by the former [16]. Thus, in the context of parent-offspring conflict, pregnancy can be seen as a struggle between the mother and the fetus in terms of the protection of their individual interests [17]. Denied pregnancies are often attended by low birth weight, prematurity, absence of nausea, and reduced abdominal swelling, all of which benefit the mother to the detriment of the fetus. The passivity on the part of the fetus can be seen as a form of its cooperation with the mother who is unwilling to incur costs to invest in the development of the fetus [17]. In such cases, while mothers bear only a fraction of the costs typically associated with pregnancy, the fetuses are compelled to endure reduced energy intake and significantly decreased antenatal protection (e.g. from injuries, food teratogens.) [17].

While there are several published case reports involving pregnancy denial (e.g. [18–20]), there is no available explanation as to the possible reasons behind this phenomenon. None of the previous studies involved a detailed postpartum follow-up of cryptic pregnancy, which our study managed to accomplish given that the patient (henceforth referred to as NN) was a participant of the RiPoD (Risk of Postpartum Depression) study (e.g. [21, 22]) and was, therefore, included in a 12-week postpartum follow-up, which involved a very close observation, made at several time points, of mood, stress levels and mother-to-child attachment. In addition, her postpartum follow-up was compared to that of the average study participant. Finally, NN’s case was discussed within the frame of available literature on pregnancy denial.

## Case presentation

This case report involves pregnancy denial in an otherwise mentally and physically healthy 38-year-old woman of German nationality who experienced a sudden delivery of a female baby. Up to the moment of the delivery, NN was not aware of being pregnant. The evening before the delivery, she had to use the bathroom frequently and experienced pain during urination, which led her to opt for pain medication. A sharp pain in the back woke her up at night, making her cry out. She needed help getting out of bed with her husband having to call for an ambulance because she could not walk to the car to be driven to the hospital. While he was on the phone and they were on their way out, she felt a tugging sensation in her abdomen and noticed the head of the baby between her legs. The baby slipped out and fell on the floor, the sound of which traumatized her a little, but otherwise she was fine during the unusual delivery. An ambulance brought NN and the infant to the hospital along with the placenta. As a precaution, the infant was taken to a pediatric ward, although there were no signs of postpartum adaptation problems. The newborn had normal weight (3220 g), and mother and child were doing extremely well, which led to their discharge within three days. NN did not have any significant birth-related injuries apart from a small tear, which she did not want to have stitched. Retrospectively, NN states that she did not experience any contractions and her water did not break. She reported feeling well both physically and mentally following the delivery. Her older children, a boy and a girl who had been born spontaneously in a different hospital, were around 8 and 11 years of age at the time, with the family planning having been complete. Nevertheless, she did not have any difficulty adjusting to the new, unexpected situation.

### Socioeconomic and medical characteristics of NN

NN has a secondary school degree and a professional training certificate. At the time of this childbirth, she had been married to her husband for 9 years having had two previous children with him. The average household income is about 30–50,000 euros per year. NN is a smoker (10–15 cigarettes per day), drinks alcohol occasionally, but does not use other drugs or medications. Throughout the denied pregnancy, she smoked and drank, and took oral contraception, which she had been doing since 2011. She reported regular withdrawal bleeding from the birth-control pill following delivery.

At the time of childbirth, NN’s BMI was approximately 35 (height 1.70 m, weight 103 kg). Although she reported being always overweight, she had been mentally and physically healthy, with no personal or family history of psychiatric disorder.

During her pregnancy, her mother had asked her if she had gained weight, and NN herself had noticed some clothes getting tighter (which she had attributed to washing them too hot). NN reported that during her previous pregnancies she had either maintained her normal weight or lost weight. During her second pregnancy, she had experienced continuous vaginal bleeding from the 11th week on. Throughout the entire unnoticed pregnancy, NN had not sensed any movements by the child. She had continued to do the requisite amount of physical work as a firefighter, repeatedly putting weight on her stomach or lying on it, to which the child had allegedly shown no reaction.

Over the course of the pregnancy, NN underwent several medical procedures. An outpatient surgery was performed under anaesthesia to remove abscesses from different parts of her body. For subsequent abscess treatment, she had come to the UKA, where she was prescribed medicine that she did not take. After a work-related accident, an ultrasound was performed on the knee and the side of the torso, as well as an X-ray of the knee and foot, both at an orthopaedist’s practice. Because of her back pain, she had gone to the emergency room of the local hospital and got an X-ray of the spine, which detected a “twisted” vertebra in her lower back. None of the doctors had suspected NN to be pregnant.

According to NN, immediately after the child’s birth, the father of the child had taken it upon himself to make all necessary arrangements while mother and child were still in the hospital. During the final interview (see RiPoD study), NN gave her husband a score of 1 (top score) regarding support at home. She reported that he had taken time off from work in order to be able to stay at home to help her get accustomed to the new situation.

The child is now 3 ½ years old and in kindergarten. NN reports that the child is developing normally, except for a delay in speech development, which was pointed out by the doctors. Notably, there are members in NN’s family with learning disabilities. According to the doctors’ reports, the delay in the child’s language development was severe, although, admittedly, it did not worry NN.

Her current life situation involves taking care of a newly paraplegic mother, which she reports being able to cope with well without experiencing any additional stress.

NN has no explanation for why she did not notice the pregnancy.

### RiPoD study

NN was a participant of the RiPoD study. All participants of study were recruited in the Department of Gynecology and Obstetrics at the University Hospital Aachen within 1–6 days of childbirth. Women with current depression or any other manifest psychiatric condition at the moment of recruitment, based on a clinical interview, were excluded from the RiPoD study. In addition, only mothers of healthy children (determined by the routine German Child Health tests [U2] conducted within the first 3–10 days of life) were included. In addition to clinical-anamnestic screenings (demographic information, information about the pregnancy as well as individual and family psychiatric history), all participants completed the Stressful Life Events Questionnaire (SLESQ; [23]) which includes possible encounters with 11 traumatic events. Symptoms of premenstrual syndrome (PMS) were assessed with the Premenstrual Tension Syndrome Scale (PTSS; [24]). As part of the RiPoD study, all participants had to complete the Edinburgh Postnatal Depression Scale [25], a 10-item self-report to help assess depressive symptomatology in the postpartum period, immediately after childbirth and every 3 weeks for 12 weeks postpartum. The Maternal Postnatal Attachment Scale (MPAS; [26]), a 19-item self-report measure of attachment quality, hostility and pleasure in interaction, and the Perceived Stress Scale (PSS; [27]) were evaluated at 3, 6, 9, and 12 weeks postpartum. At 3 weeks postpartum, the experience of baby blues symptoms was assessed with both a self-report and a cut off score of > 10 on the Maternity Blues Questionnaire (MBQ) [28, 29]. Every second day during the study period, the participants were asked to rate their stress and mood level on a response scale from 1 (indicating low levels of stress/mood) to 10 (high levels of stress/mood). Remote assessments were sent via e-mail. To ensure that the participation was closely monitored, a reminder was sent via e-mail in case three consecutive assessments were missed [22, 30]. Twelve weeks after childbirth, the participants were invited to a final semi-standardized clinical interview for the final diagnosis (postpartum adjustment disorder, postpartum depression) by an experienced psychiatrist or psychologist. The study protocol was drafted in accordance with the Declaration of Helsinki and approved by the Institutional Review Board of the Medical Faculty, RWTH Aachen University (EK 208 − 15).

For direct comparisons between NN’s postpartum course and that of new mothers in general, we selected four control subjects (CS) from the RiPoD study who were not depressed during the follow-up phase of the study and had similar sociodemographic backgrounds: married or in a relationship, 3 children in total, 10 years of education, middle socioeconomic class, and no psychiatric history.

Additionally, we have provided information on the mean postpartum mood and stress score, EPDS mean score, PSS mean score, and MPAS mean score for 558 study participants of the total sample (recruited between November 2015 and November 2021) who were not depressed during the postpartum period. The group’s sociodemographic and clinical history data are provided in the Supplement.

### Comparison between NN and the four matched controls based on sociodemographic and clinical-anamnestic data

The four matched control subjects were between 31 and 36 years of age. The sociodemographic and clinical-anamnestic data are shown in the supplementary Table S1. With the exception of CS_1, who had a preterm birth (36 weeks’ gestation) and whose baby was transferred to an infant unit, the other three women delivered healthy mature infants. CS_1 and CS_4 reported a family psychiatric history (first-degree family), and CS_2 reported two stressful life events. NN and CS_2 reported suffering from symptoms of premenstrual syndrome. With the exception of NN, none of the women reported a subjectively traumatic birth experience, and one (CS_1) reported having had baby blues.

### Comparison between NN and four matched controls based on the postpartum follow-up

**Fig. 1:**
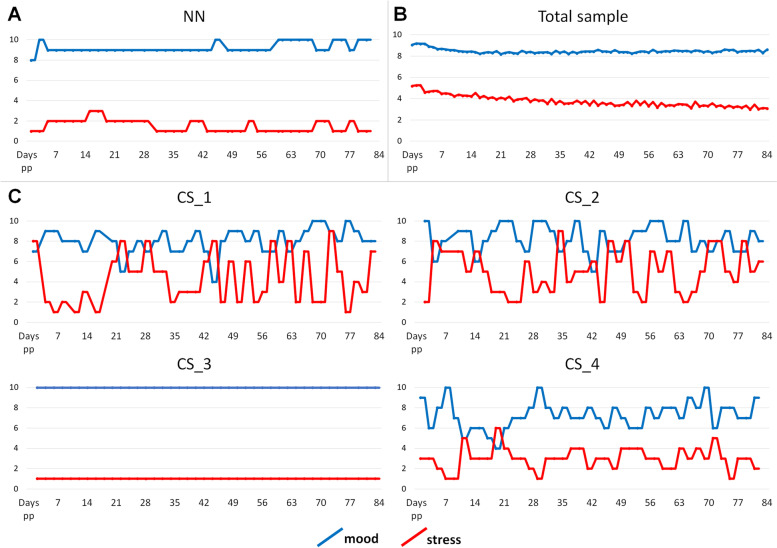
Mood and stress scores throughout the study period (84 days) of **A** NN, **B** averaged across the total RiPoD study sample, and **C** of four matched controls (CS_1, CS_2, CS_3, CS_4)

Mood and stress levels were queried every other day for 84 days, resulting in 42 expected responses per participant. NN gave 40 responses and had a mood sum score of 370 (M = 9.25, SD = 0.49) and a stress sum score of 60 (M = 1.50, SD = 0.60) (see Fig. 1 A). Of the four matched controls, only CS_3 reported extremely high mood and low stress throughout the study period (see Fig. 1C). The other three control subjects (CS_1, CS_2, CS_4) reported extremely fluctuating mood and stress scores (see Fig. 1C).

NN reported EPDS scores of 0 at all measurement time points, a PSS score of 3 at T1 and 0 at T2, T3 and T4, and MPAS scores in the range of 87 to 94 throughout the study period. Except CS_2 and CS_4, who reported slightly elevated EPDS scores of 9 and 6, respectively, at the time of childbirth, the subsequent time points as well as the scores of CS_1 and CS_3 were similarly low as those of NN. CS_1 did not provide PSS scores, CS_2 and CS_3 reported similarly low PSS scores as NN, and CS_4 reported very high PSS scores throughout the study period (see supplementary Table S1).

### Comparison between NN and 558 participants of the RiPoD study

The sociodemographic and clinical-anamnestic data of the 558 non-depressed RiPoD study participants are provided in the supplementary Table S2. The mean age of the women was 32.41 years (SD = 4.45).

In terms of mood scores, NN was not much higher than average as the study sample included only non-depressed postpartum women who also reported high mood scores. Using the data of those who provided between 35 and 42 responses at the follow-up (*n* = 451), the mean mood score was found to be 8.46 (SD = 0.19) and the mean stress score was 3.76 (SD = 0.52) (see Fig. 1 B). Within the first two days of delivery, NN reported a stress score of 1 (the minimal possible level of stress), a score otherwise reported by only 19 women (4.21%) of the total sample. However, of these women, 14 (73.68%) reported highly fluctuating stress values with maximum values up to 10 in the following 12 weeks. The maximum stress value reported by NN was 3. In the total sample, only 15 women (3.32%) reported the same maximum value.

**Fig. 2:**
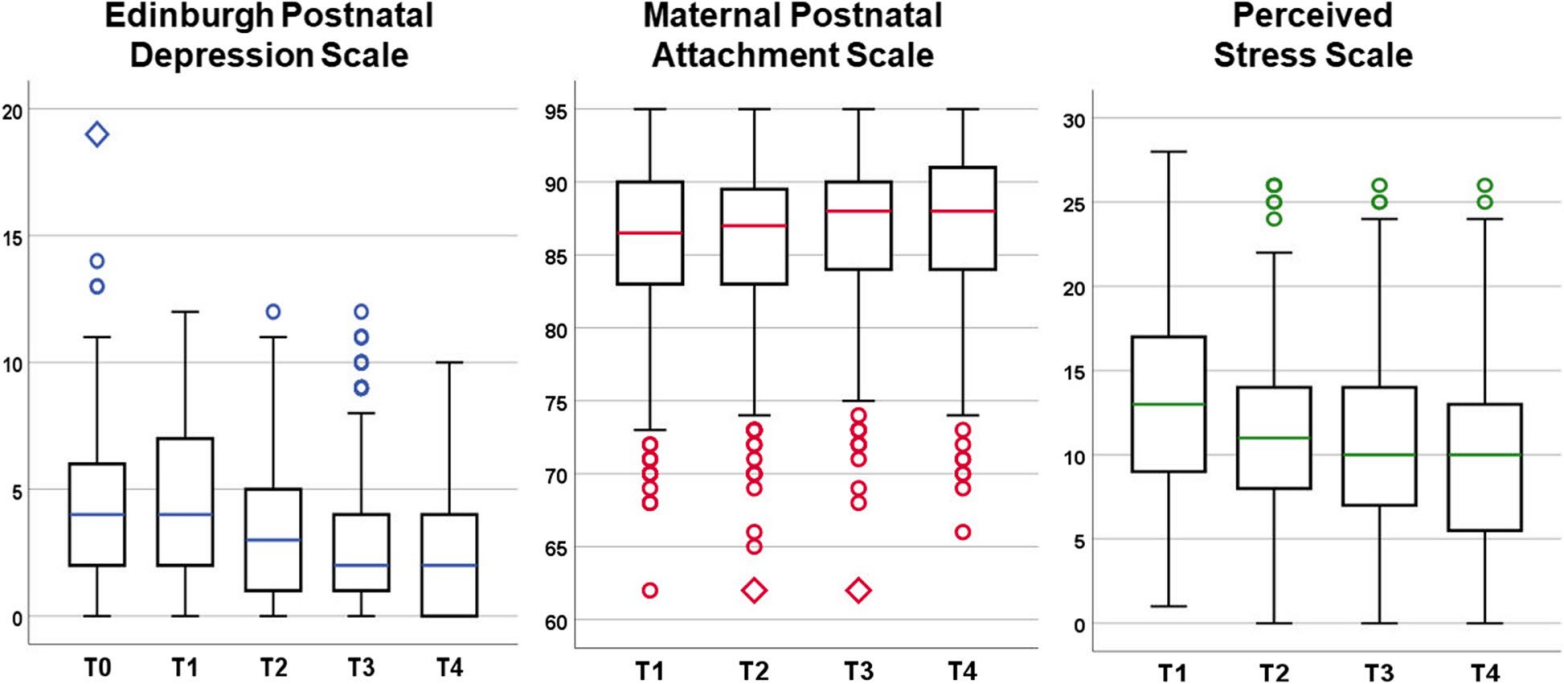
Box plots of the Edinburgh Postnatal Depression Scale, the Perceived Stress Scale, and the Maternal Postnatal Attachment Scale scores of the non-depressed study sample (*n* = 558). Lower and upper box boundaries represent the 25th and 75th percentiles, respectively, lower and upper whiskers 10th and 90th percentiles, respectively. Circles above and below the whiskers indicate outliers

Mean scores of the EPDS, the MPAS and the PSS for the study sample are provided in the supplementary Table S2 and can be seen in Fig. 2.

There were no other women in the entire postpartum sample (*n* = 558) who reported a PSS score of 0 at T2, T3, and T4 throughout the follow-up period, regardless of the score at T1. Only four women (0.72%) reported scores below 3 at all time points.

Although there is no cut-off score for low stress in the PSS, we used the 10th percentile to define low stress, resulting in a cut-off score of 7 at T1, a score of 5 at T2, a score of 4 at T3 and a score of 3 at T4. 13 women (2.33%) reported these or lower values at the respective time points.

Only nine women (1.61%) reported EPDS scores of 0 at all measurement time points. Of these participants, only three reported similarly low PSS scores as NN, with scores ranging from 0 to 3 at different time points. However, the remaining 6 women reported elevated PSS scores at all measurement time points (T1: 5.33 ± 3.14; T2: 3.5 ± 2.81, T3: 4.67 ± 2.49; T4: 4.5 ± 3.15), ranging from 0 to 11.

There were 26 participants (4.66%) who reported the same or higher MPAS scores as NN at the respective time points. However, the EPDS scores of these women ranged from 0 to 13 (T0: 3.46 ± 3.26; T1: 2.81 ± 2.74; T2: 1.58 ± 1.96; T3: 1.19 ± 1.71; T4: 1.3 ± 2.07) and the PSS scores from 0 to 23 (T1: 10.05 ± 4.70; T2: 8.6 ± 4.75; T3: 7.65 ± 4.68; T4: 6.25 ± 5.42).

## Discussion

NN was a participant in the ongoing project related to early recognition of postpartum disorder, which entails continuous monitoring of stress, mood level and mother-to-child attachment. Given the unusual circumstances involving the delivery of NN’s child, we sought to compare her postpartum course with that of other participants matched on the basis of socioeconomic characteristics. In comparison to other participants, NN was not found to have disturbed mood at any time point following childbirth. Her stress level remained remarkably low throughout the entire 12-week observation period. Compared to her, other participants reported a large variation in mood and stress levels, which is understandable given the challenges of having a new baby. In view of the circumstances involving NN’s delivery, her stress level (1, the minimal possible level of stress) on the day of delivery was also quite extraordinary given that she had no time to prepare herself for the new situation. According to her report, she did not need any time to adjust herself to the fact of suddenly having a third child.

We sought to understand the possible reasons behind NN’s denial of pregnancy based on the available literature. One of the most consistent aspects of this phenomenon appears to be the lack of awareness of any symptoms of pregnancy. NN’s report is in line with this fact. Nausea and amenorrhea are widely considered to be the primary cues to pregnancy [5, 31]. In cryptic pregnancy, the physiological symptoms (nausea, amenorrhea, increased breast size, weight gain and abdomen swelling) are often either absent or greatly reduced [4, 5]. Paradoxically enough, menstruation-like bleeding is also reported in cryptic pregnancy [2, 5]. Nausea is found to be suffered most rarely by women who are unaware of pregnancy until delivery [6]. This suggests that woman with insufficient cues are unlikely to realize they are pregnant. In addition to the lack of symptoms, NN was taking contraceptive pills throughout her pregnancy, which likely contributed to her vaginal bleeding and her false sense of not being pregnant. An association between oral contraceptive and pregnancy denial has been reported in several other cases [4]. Interestingly, that NN was pregnant was not noticed by her family members either, which is also typical of the phenomenon of pregnancy denial [2]. Based on her own deep-rooted conviction of not being pregnant, the pregnant woman succeeds including people around her into the denial of pregnancy mindset, resulting in their physicians, family members and even sexual partners being unaware of their gravid state [2, 32, 33]. In some cases, the doctors of the pregnancy deniers interpret complaints like noise and belly pain as somatic problems such as spine prolapse, ascites, etc. [2, 32, 33]. That was also the case with NN, who had been to the hospital twice, and even got an X-ray done on account of the back pain. It has been suggested that the pregnancy denier’s auto-suggestive wishful notions lead to suggestive confirmation by the physician’s misdiagnosis, which contributes to a further denial of pregnancy [32].

Notably, NN did not belong to the typical group of pregnancy denying women, who tend to be younger, less educated, and are in a more precarious life situation, and are frequently single [4]. At the time of this pregnancy and delivery, she was in a stable relationship, having already given birth to two other children. However, research has revealed no unique typology of the ‘pregnancy denier’ [3, 34].

Pregnancy denial can jeopardize the health of the mother and the newborn, particularly because of a late or non-existent obstetrical follow-up or continuation of teratogen habits (tobacco, alcohol, etc.) [31, 34]. Low birth weight has been found to be a salient feature of cryptic pregnancy, with the proportion of viable neonates weighing less than 2500 g being very high in one German [6] and one Austrian [5] sample compared to the 7% in the general population [35]. This, however, is not corroborated by all reports, and a more recent study has found no relevant differences [4]. In the case of NN, there were some risks with respect to the child, particularly given that the mother continued to smoke and drink. In addition, she continued working as a firefighter, admittedly lifting heavy weights. The child, however, did not show any sign of growth retardation or adaptation problems at the time of delivery.

Another risk factor associated with cryptic pregnancy is the mother’s potential psychological distress, or harm caused by the mother to the newborn, which may result in neonaticide [13]. With NN, however, neither problem was apparent. Compared to other mothers, she reported to have high (above-average values) mother-child attachment scores with only 4.6% of other women reporting the same or higher scores. In addition, NN reported to have no disturbance in daily mood and stress levels. Her daily stress scores were also unaffected, showing no disturbances over the 12 postpartum weeks. Compared to the total sample, NN did not show any natural ups and downs in mood and stress, which was also the case for only 3.32% of the other women. The remote assessment of stress every three weeks (PSS score) is also characterized by a complete absence of stress, which was similarly reported by only 0.72% of all women in the sample. Baby blues are common in young mothers, affecting at least 55% of them [36]. In the non-depressed postpartum sample, 36% experienced baby blues, while NN reported no symptoms of the condition. Also, she did not have any history of psychiatric disorder, trauma or stressful life events, thus lacking any significant psychiatric risk factor. Additionally, NN reported no problems or conflicts in her personal circumstances or those involving her family. Remarkably, however, she showed persistent resilience to the postpartum follow-up in comparison to the average study participant. Only 1.61% of participants reported a complete lack of any symptoms in the EPDS scores. We do not have any reason to believe that NN was not honest while responding to the questionnaires.

In sum, the most obvious reason behind NN’s pregnancy denial seems to be the lack of symptoms typically associated with pregnancy. What is not clear, however, is why the pregnancy-related symptoms were absent. With the age of the mother as well as with the number of previous pregnancies, the probability of detecting a pregnancy before the 6th week increases [37]. In general, women who want to have children pay more attention to the signs and symptoms of pregnancy, which is unlikely to be the case for women who are convinced they cannot become pregnant because they rely on contraceptives. Most unintended pregnancies among contraceptive users occur when contraceptives are used inconsistently or incorrectly [38], but also when women are overweight [39].

In such cases, a woman’s conviction about not being pregnant likely overrides the possible signs of pregnancy. Some personality traits can also contribute to situations like this. For instance, we saw very little variation in NN’s stress and mood levels, which may account for a somewhat reduced self-perception or self-awareness. It is through self-awareness that one is conscious of one’s own character, feelings, motives, and desires. A diminished sense of it can lead one to ignore one’s private needs, and being unaware of one’s own feelings, which may not necessarily be a negative trait, and, as presumably in the case of NN, can also lead to emotional-behavioral resilience. NN demonstrated an incredible degree of it in dealing with the situation she suddenly found herself in with a new child. However, NN reported the experience of delivery to be traumatic given that the child had fallen to the floor. In addition to working as a firefighter, which is hardly typical of a woman, and caring for her family, she was looking after her paraplegic mother, apparently unaffected by the illness and easily coping with the attendant challenges. Her child’s developmental delay at age 3, which was severe according to the doctors, did not appear to trouble her either. Thus, it may be safe to assume that a reduced self-perception was likely at the core of NN’s coping strategy, which did not allow the circumstances to get the better of her.

One limitation of the present report is the paucity of information regarding the progression of childbirth from an obstetric point of view. This is because no medical professional was present during delivery and all relevant information is based solely on the self-reports of NN, who is not knowledgeable about medical matters and her accounts of the events surrounding the delivery are also not entirely reliable given the stressful state of her mind at the time. Understandably, due to the sensitive nature of the subject, NN also deliberately withheld some private information. In addition, there are no relevant details about NN’s previous pregnancies.

Although the case report involves a comparison with a larger group of postpartum women, no conclusion can be drawn about postpartum adaptation following cryptic pregnancies. Thus, the presented case remains a single case, precluding any generalization with regard to denied pregnancies. Although NN was found to behave unusually compared to other postpartum women who participated in the study, no conclusion can be drawn about how women would behave in similar situations.

All this notwithstanding, a profound strength of this case report, besides the close monitoring of the patient for 12 weeks, is based on our ability to contact and follow-up with the patient 3 years after the childbirth and obtain information about the family’s development.

Based on our observation of NN, we conclude that the reasons behind a pervasive denial of pregnancy can vary depending on personal situations. In some cases, a pervasive denial may be a normal variation without links to any psychological or physiological condition. Perhaps there is no postpartum behavior that can be identified as “typical” of a cryptic pregnancy, because there is no particular “type” of women who are more likely than others to be affected by that phenomenon. Even after a cryptic pregnancy, postpartum adaptation and behavior will likely depend on the mother’s personal circumstances. More research involving bigger cohorts is necessary to understand if there are any “trait” or “state” characteristics that are affected by this unusual phenomenon.

## Supplementary Information

**Additional file 1: Table S1.** Sociodemographic and clinical-anamnestic data of NN and four matched control subjects of the RiPoD study. **Table S2.** Socioeconomic characteristics of the entire study sample (*n* = 558).

## Data Availability

The data presented in this study are available on reasonable request from the corresponding author. The data are not publicly available due to privacy restrictions.

## Abbreviations

CS: Control subjects
EPDS: Edinburgh Postnatal Depression Scale
MBQ: Maternity Blues Questionnaire
MPAS: Maternal Postnatal Attachment Scale
PMS: Premenstrual syndrome
PSS: Perceived Stress Scale
PTSS: Premenstrual Tension Syndrome Scale
RiPoD study: Risk of postpartum depression study
SLESQ: Stressful Life Events Questionnaire
T0: 1-6 days after childbirth
T1: 3 weeks after childbirth
T2: 6 weeks after childbirth
T3: 9 weeks after childbirth
T4: 12 weeks after childbirth
