# Supplementary material for: A case report involving the experience of pervasive pregnancy denial: detailed observation of the first 12 postpartum weeks
Source: BMC Psychiatry. 2022 Dec 9;22:774. doi: 10.1186/s12888-022-04377-1 (PMC9732985; doi:10.1186/s12888-022-04377-1)
Supplement: Supplementary file 1 — Additional file 1: Table S1. Sociodemographic and clinical-anamnestic data of NN and four matched control subjects of the RiPoD study. Table S2. Socioeconomic characteristics of the entire study sample (n = 558). [file 12888_2022_4377_MOESM1_ESM.docx]

*Supplementary Information for*

**A case report involving the experience of pervasive pregnancy denial: detailed observation of the first 12 postpartum weeks**

Chechko N, Losse E, Stickel S

Table S1. Sociodemographic and clinical-anamnestic data of NN and four matched control subjects of the RiPoD study.

|  | NN | CS_1 | CS_2 | CS_3 | CS_4 |
| --- | --- | --- | --- | --- | --- |
| **Age** | 38 | 36 | 31 | 33 | 35 |
| **Intention to breastfeed at T0** | no | yes | no | yes | yes |
| **Breastfeeding after 12 weeks pp** | no | yes | no | yes | yes |
| **Birth mode** | vaginal | vaginal | c-section | vaginal | vaginal |
| **Weeks of gestation** | - | 36+0 | 39+2 | 40+0 | 39+2 |
| **Infant's relocation to special ward** | yes | yes | no | no | no |
| **Gender of the child** | female | female | male | male | female |
| **Child's birthweight (gram)** | 3220 | 2380 | 3420 | 3100 | 3440 |
| **Familial psychiatric history** | no | yes | no | no | yes |
| **Number of stressful life events** | 0 | 0 | 2 | 0 | 0 |
| **Support at home (grade 1-6)** | 1 | - | 2 | 2 | 3 |
| **Premenstrual symptom severity** | severe | - | mild | none | none |
| **Subj. experience of birth-related trauma** | yes | no | no | no | no |
| **Baby blues in the first 2 weeks pp** | no | yes | no | no | no |
| **EPDS T0** | 0 | 2 | 9 | 1 | 6 |
| **EPDS T1** | 0 | 2 | 1 | 0 | 2 |
| **EPDS T2** | 0 | 1 | 0 | 1 | 2 |
| **EPDS T3** | 0 | 0 | 0 | 0 | 1 |
| **EPDS T4** | 0 | 0 | 0 | 0 | 1 |
| **MPAS T1** | 91 | 89 | 91 | 91 | 86 |
| **MPAS T2** | 94 | 91 | 91 | 91 | 85 |
| **MPAS T3** | 89 | 94 | 91 | 95 | 84 |
| **MPAS T4** | 87 | 91 | 91 | 91 | 83 |
| **PSS T1** | 3 | - | 4 | 3 | 17 |
| **PSS T2** | 0 | - | 3 | 1 | 17 |
| **PSS T3** | 0 | - | 9 | 0 | 17 |
| **PSS T4** | 0 | - | 4 | 0 | 10 |

Note. T0: 1-6 days after delivery; pp: postpartum. EPDS: Edinburgh Postnatal Depression Scale; MPAS: Maternal Postnatal Attachment Scale; PSS: Perceived Stress Scale; T0: 1-6 days after childbirth; T1: 3 weeks postpartum; T2: 6 weeks postpartum; T3: 9 weeks postpartum; T4: 12 weeks postpartum.

Table S2. Socioeconomic characteristics of the entire study sample (n = 558).

|  | Mean (SD) | Range | % |
| --- | --- | --- | --- |
| Age | 32.41 (4.45) | 18 - 46 |  |
| Family status (with partner) |  |  | 98 |
| Marital status (married) |  |  | 75.5 |
| Total number of children | 1.61 (0.77) | 1 - 5 |  |
| Intention to breastfeed at T0 |  |  | 89.7 |
| Breastfeeding after 12 weeks pp |  |  | 77.8 |
| Highest degree of education |  |  |  |
| < 9 years |  |  | 3.8 |
| 10 years |  |  | 13.5 |
| > 13 years |  |  | 82.7 |
| Income |  |  |  |
| < 20k € |  |  | 9.5 |
| 20k - 50k € |  |  | 30.9 |
| > 50k € |  |  | 59.7 |
| Birth mode |  |  |  |
| Vaginal |  |  | 57.3 |
| Ventouse |  |  | 5.7 |
| C-section |  |  | 25.9 |
| Emergency section |  |  | 11.1 |
| Complications at birth |  |  | 26 |
| Complication during pregancy |  |  | 42.7 |
| Days of gestation | 273.71 (11.87) | 211 - 294 |  |
| Infant's relocation to special ward |  |  | 23.8 |
| Child’s birthweight (gramm) | 3357.22 (510.27) | 1450 - 5010 |  |
| Psychiatric history |  |  | 13.5 |
| Previous postpartum depression |  |  | 2.5 |
| Family psychiatric history |  |  | 22.6 |
| Number of stressful life events | 0.88 (1.32) | 0 - 9 |  |
| At least on stressful live event |  |  | 45.4 |
| Support at home (grade 1-6) | 1.78 (0.85) | 1 - 6 |  |
| Premenstrual syndrome severity |  |  |  |
| None |  |  | 53.7 |
| Mild |  |  | 35.6 |
| Severe |  |  | 10.7 |
| Experience of birth-related trauma |  |  | 9.9 |
| Baby Blues in the first 2 weeks pp |  |  | 36.5 |
| EPDS T0 | 4.21 (3.04) | 0 - 19 |  |
| EPDS T1 | 4.49 (2.88) | 0 - 12 |  |
| EPDS T2 | 3.35 (2.68) | 0 - 12 |  |
| EPDS T3 | 2.8 (2.64) | 0 - 12 |  |
| EPDS T4 | 2.48 (2.36) | 0 - 10 |  |
| MPAS T1 | 85.79 (5.41) | 62 - 95 |  |
| MPAS T2 | 86.05 (5.34) | 62 - 95 |  |
| MPAS T3 | 86.53 (5.19) | 62 - 95 |  |
| MPAS T4 | 86.98 (4.93) | 66 - 95 |  |
| PSS T1 | 13.32 (5.49) | 1 - 29 |  |
| PSS T2 | 11.6 (5.037) | 0 - 26 |  |
| PSS T3 | 10.42 (5.12) | 0 - 26 |  |
| PSS T4 | 9.75 (5.32) | 0 - 26 |  |

Note. EPDS: Edinburgh Postnatal Depression Scale; MPAS: Maternal Postnatal Attachment Scale; PSS: Perceived Stress Scale; T0: 1-6 days within childbirth; T: 3 weeks postpartum (pp); T3: 9 weeks pw T4: 12 weeks pp.
